# Biochemical and Molecular Characterization of PvNTD2, a Nucleotidase Highly Expressed in Nodules from *Phaseolus vulgaris*

**DOI:** 10.3390/plants9020171

**Published:** 2020-02-01

**Authors:** Gregorio Galvez-Valdivieso, Elena Delgado-Garcia, Mercedes Diaz-Baena, Oscar Montaño, Francisco A. Quiles, Manuel Pineda, Pedro Piedras

**Affiliations:** Departamento de Botánica, Ecología y Fisiología Vegetal. Plants Molecular Physiology and Biotechnology Group, Campus de Rabanales, Edif. Severo Ochoa, Universidad de Córdoba, 14071-Córdoba, Spain; b32gavag@uco.es (G.G.-V.); b22degae@uco.es (E.D.-G.); b42dibam@uco.es (M.D.-B.); oscarmonra92@gmail.com (O.M.); b42quluf@uco.es (F.A.Q.); bb1piprm@uco.es (M.P.)

**Keywords:** *Phaseolus vulgaris*, ureide synthesis, phosphatase, nucleotidase, nucleoside monophosphate, nodules

## Abstract

Nucleotides are molecules of great importance in plant physiology. In addition to being elementary units of the genetic material, nucleotides are involved in bio-energetic processes, play a role as cofactors, and are also components of secondary metabolites and the hormone cytokinin. The common bean (*Phaseolus vulgaris*) is a legume that transports the nitrogen fixed in nodules as ureides, compounds synthetized from purine nucleotides. The first step in this pathway is the removal of the 5’-phosphate group by a phosphatase. In this study, a gene that codes for a putative nucleotidase (*PvNTD2*) has been identified in *P. vulgaris*. The predicted peptide contains the conserved domains for haloacid dehalogenase-like hydrolase superfamily. The protein has been overexpressed in *Escherichia coli,* and the purified protein showed molybdate-resistant phosphatase activity with nucleoside monophosphates as substrates, confirming that the identified gene codes for a nucleotidase. The optimum pH for the activity was 7–7.5. The recombinant enzyme did not show special affinity for any particular nucleotide, although the behaviour with AMP was different from that with the other nucleotides. The activity was inhibited by adenosine, and a regulatory role for this nucleoside was proposed. The expression pattern of *PvNTD2* shows that it is ubiquitously expressed in all the tissues analysed, with higher expression in nodules of adult plants. The expression was maintained during leaf ontogeny, and it was induced during seedling development. Unlike PvNTD1, another NTD previously described in common bean, the high expression of *PvNTD2* was maintained during nodule development, and its possible role in this organ is discussed.

## 1. Introduction

Nucleotides are crucial compounds for plant metabolism and development. Purine and pyrimidine nucleotides are components of nucleic acids and, therefore, essentials for genetic information storage and for the machinery required for protein synthesis. Nucleotides are also components of vitamins and coenzymes and are important as energy donor molecules [1]. Purine nucleotides have a relevant role as precursors of ureides in the ureidic legumes such as soybeans and the common bean. These plants transport most of the nitrogen fixed in the nodules to the upper parts of the plants as ureides; therefore, purine nucleotide metabolism is crucial in the nodules of these plants. Ureides have been implicated in other processes with high nutrient transport requirements, such as seedling development [2,3] and leaf senescence [4]. Studies performed in the model plant *Arabidopsis* suggest a function for ureides in response to abiotic stresses such as dark stress [5], drought [6], and salt stress [6,7]. The accumulation of ureides in response to stress might suggest a role for these compounds as protectors against the effects of reactive oxygen species [8], and recently we have described a relationship between ureide metabolism and antioxidant activities in legume seedlings [9].

Nucleotide metabolism can be divided into three parts: de novo synthesis, salvage of nucleosides and nucleobases, and catabolism of purines and pyrimidines [1]. The salvage pathway can be important in tissues with low demand for purines, whereas de novo synthesis would be the main route for purine synthesis in highly purine-producing tissues as nodules [10]. The salvage and de novo synthesis pathways converge at the formation of nucleoside monophosphate. The first step in the catabolic pathway is the removal of the 5´-phosphate group catalysed by a phosphatase that hydrolyses the nucleotides into nucleosides. However, it is unclear if this step is catalysed only by one enzyme or if the dephosphorylation reactions of different nucleotides are facilitated by several enzymes. Furthermore, it is also unclear if this step is catalysed by a nonspecific acid phosphatase (EC 3.1.3.2) or a specific 5’-nucleotidase (EC 3.1.3.5). Nucleoside kinases catalyse the reverse reaction to 5’-nucleotidase, and these opposite reactions have been suggested for almost all the nucleotide–nucleoside pairs [11]. In this way, the balance between synthesis and degradation of nucleotides can be regulated by the ratio between the phosphatase and kinase activities [11]. The nucleotide pools must be adjusted to the differing needs during the phases of metabolism; therefore, regulatory mechanisms are used to coordinate the absolute and relative levels of purines and pyrimidines both between cells and between subcellular compartments, as well as the relative levels of mono-, di-, and triphosphate forms [1].

Previously, we have purified and characterised phosphatase activity from common bean seedlings with high affinity for nucleotides [12,13]. This activity is insensitive to the phosphatase inhibitor molybdate [14]. The sequence of the gene encoding the common bean nucleotidase, *PvNTD1* [13], shows similarity to the phosphatase/nucleotidase from soybean nodules described by Penheiter and colleagues [15,16], to a phosphatase gene induced by wounding in poplar [17], and to some genes encoding vegetative storage proteins with antimicrobial activity identified in *Arabidopsis* [18]. All these genes belong to the haloacid dehalogenase (HAD) superfamily of hydrolases. The family members are identified by the presence of four short motifs with conserved catalytic domain, although the overall sequence identity between HAD phosphatases is very low [19]. Although the catalytic domain is more conserved, the catalysed reaction and substrate specificity are difficult to predict and need to be determined empirically. HAD phosphatases are a very large and ubiquitous class of enzymes found in all the three superkingdoms of life, and they have attracted increased medical interest because of the involvement of some members of this family in diseases such as cancer and cardiovascular, metabolic, and neurological disorders [20]. The loss of some HAD phosphatases causes hereditary disorders in humans, disagreeing with the traditional view of this family as metabolic phosphatases with relaxed substrate specificities and with housekeeping functions [20]. The knowledge about the function of the phosphatases from this family in plants is more limited, but several HAD superfamily members have been involved in the regulation of Pi homeostasis [21,22,23].

To better understand the complex family of nucleotidases in plants, we have cloned and characterised the gene encoding a new nucleotidase member of the HAD superfamily in *Phaseolus vulgaris*. In addition, we have analysed its expression pattern in different tissues and developmental stages discussing the differences with the other member of this family previously characterised.

## 2. Results

### 2.1. Identification, Cloning, and Overexpression of PvNTD2

**Figure 1:**
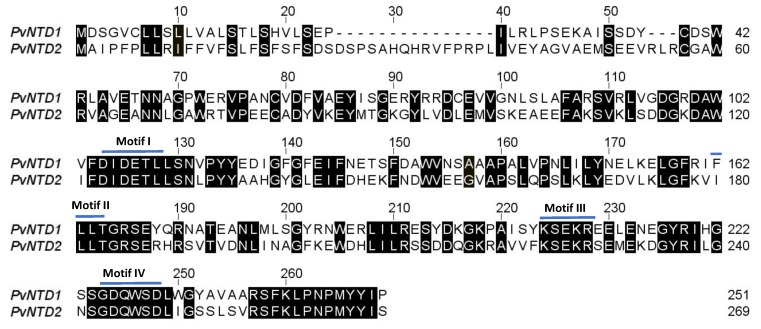
Alignment of amino acid sequences of PvNTD2 with the previously identified PvNTD1 [13]. Black boxes indicate the amino acids that are identical in both sequences. Motifs I to IV of the haloacid dehalogenase (HAD) superfamily [27] are indicated. Protein sequences were aligned using the ClustalW method.

To identify new phosphatases with high affinity for nucleotides, a tblastn using the *PvNTD1* sequence (Phvul.004G174200) was conducted in the NCBI database in the *Phaseolus vulgaris* genome [24]. This search retrieved the *PHAVU_011G182400g* gene as the sequence with the highest similarity to *PvNTD1*, so it was called *PvNTD2*. The *PvNTD2* full-length cDNA sequence is 1077 bp long, with the largest coding sequence of 810 bp encoding a 269 amino acid polypeptide with a predicted molecular weight of 30.6 kDa (Figure 1). The prediction of the subcellular location with DeepLoc [25] indicates that PvNTD2 could be an extracellular soluble protein (probability 0.73), and a sequence analysis with signalP-5.0 [26] predicts a cleavage site between position 24 and 25 (probability 0.95). After processing the signal peptide, the protein would have a molecular weight of 27.8 kDa, an isoelectric point of 5.04, and a charge of −10.11 at pH 7.0. The level of identity between PvNTD1 and PvNTD2 is 47%, and the alignment between these two sequences is shown in Figure 1. PvNTD2 contains the domains conserved for haloacid dehalogenase-like hydrolase superfamily [24] consisting of motifs I to IV (Figure 1). A tblastn search in the GENBANK database using the PvNTD2 amino acid sequence was performed to identify similar sequences. This analysis revealed the existence of similar sequences in several plant genomes (Table S1), which are annotated as acid phosphatases, but which have not been characterized yet.

**Figure 2:**
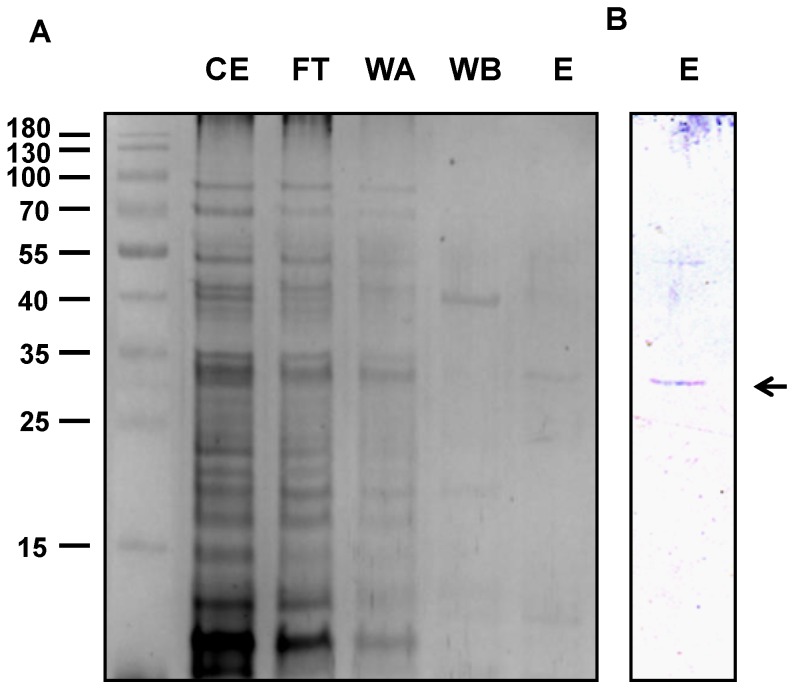
Purification of recombinant PvNTD2 from *Escherichia coli*. The purification process is indicated in Materials and Methods section. Lane CE: crude extracts, lane FT: proteins eluted from the column after loading the crude extract (unbound proteins), lane WA: proteins eluted from the column with washing buffer, lane WB: proteins eluted with washing buffer supplemented with 25 mM imidazole, lane E: proteins eluted from the column after elution with 0.2 M imidazole. (**A**). Samples were analysed by silver staining. (**B**) Samples were analysed by Western blot. The positions of the molecular weight markers are shown left of the figure.

The open reading frame of *PvNTD2* without the predicted signal peptide was amplified by PCR, cloned into the vector pET30b (+), and transformed into *Escherichia coli* BL21 (DE3) cells. The expression of the His-tagged recombinant protein was induced by IPTG, and the protein extract of these cultures was analysed by SDS-PAGE showing a band of around 32 kDa in both the soluble and the insoluble fractions, which indicates that part of the protein aggregates formed inclusion bodies. The soluble fraction was used to purify the recombinant protein by affinity chromatography with Ni sepharose (Figure 2). Apart from the expected protein, the antibodies revealed a second band with a molecular mass consistent with that of a dimer (Figure 2).

### 2.2. Protein Characterization

**Figure 3:**
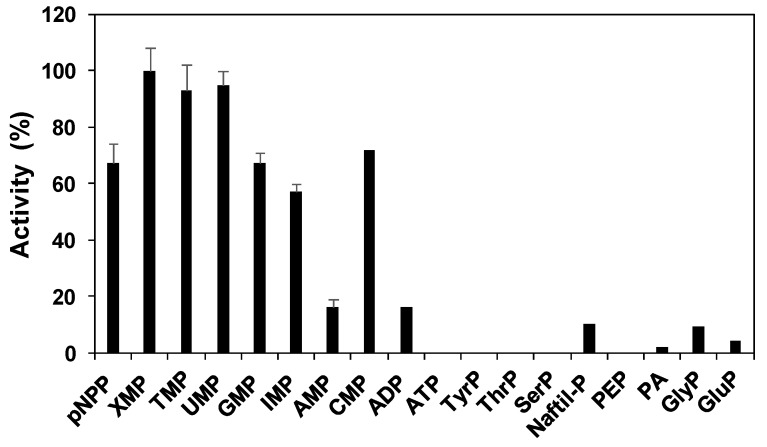
Phosphatase activity of purified PvNTD2 with several phosphorylated compounds. The activity was determined under standard assays with the compound indicated at a final concentration of 2 mM. One hundred percent corresponded to 4.1 U/ mg protein. Values are mean ± SE of three independent determinations.

The phosphatase activity was determined with several phosphorylated compounds at pH 7.0 (Figure 3). The purified protein showed activity mainly with nucleoside monophosphates as substrates, whereas the activity determined with other phosphorylated compounds was minimal except for pNPP, an artificial compound used routinely to determine phosphatase activity. The activity was maximal when assayed with XMP, UMP, and TMP as substrates, and the specific activity of the purified protein was 4.1, 3.9, and 3.8 U/mg, respectively. The activity with CMP, GMP, and IMP was slightly lower than that with the previously indicated substrates, with activity values of 3.0, 2.7, and 2.3, respectively, corresponding to 75%–60% of maximal activity. With AMP as substrate, the determined nucleotidase activity was only 0.7 U/mg, which was the lowest among all the assayed nucleosides monophosphate.

The K_m_ and V_max_ values were calculated by Hanes–Woolf plots with nucleotides with high activity as well as with AMP (Table 1). The K_m_ value with AMP as substrate (0.011 mM) was lower than that of the other nucleotides assayed, which ranged from 0.039 mM for UMP to 0.095 mM for XMP. The catalytic efficiency calculated as V_max_/K_m_ was higher with AMP than with the other nucleotides.

**Figure 4:**
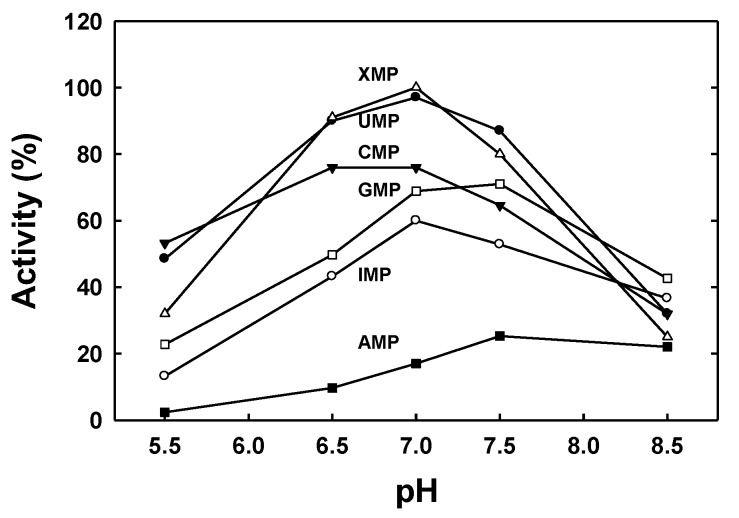
Effect of pH on the activity of PvNTD2 with several nucleotides as substrates. The activity was determined in 50 mM MES-Tris buffer at the indicated pH values.

The optimum pH with all the substrates analysed was between 7 and 7.5, with a marked decrease in activity at pH values below 6.5 or above 7.5, with the exception of AMP (Figure 4). With AMP as substrate, the nucleotidase activity of the purified protein was very similar at pH 8.5 and 7.5, and AMP was the only substrate with which the activity at pH 8.5 was higher than that at pH 7 (Figure 4).

**Figure 5:**
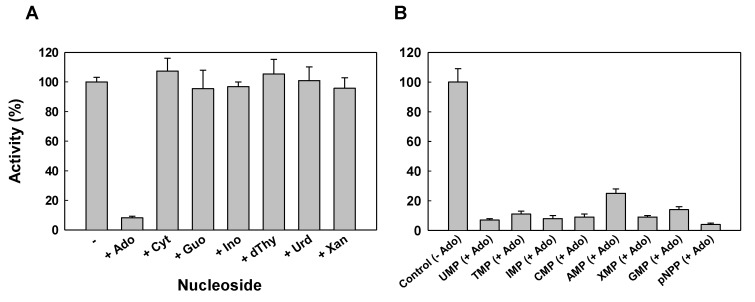
Effect of nucleosides on the activity of PvNTD2. (**A**) Phosphatase activity was determined with 2 mM UMP as substrate in the absence of nucleoside (-) or in the presence of 0.2 mM adenosine (+Ado), cytosine (+Cyt), guanosine (+Guo), inosine (+Ino), thymidine (+dThy), uridine (+Urd), or xanthosine (+Xan). The activity in the absence of nucleoside was considered as 100%. (**B**) Effect of adenosine on the activity of PvNTD2 with several nucleotides as substrates. Phosphatase activity was determined with 2 mM of nucleotide indicated in the absence or presence of 0.2 mM adenosine. The activity with every nucleotide in the absence of nucleoside was normalised as 100% (control), and the corresponding activity in the presence of adenosine is represented for each nucleotide as substrate. Values are mean ± SE of three independent determinations.

The effect of several compounds on the phosphatase activity of the purified protein was determined at pH 7. The activity was insensitive to the inhibitors of unspecific acid phosphatases such as vanadate, molybdate, tartrate, or fluoride at a final concentration of 1 mM. Free phosphate (0.1 mM), a product of the reaction, was assayed in the reaction with pNPP as substrate, and no effect was observed in the activity. The phosphatase activity was determined as well in the presence of several nucleosides, the other products of the reaction, at a final concentration of 0.2 mM in the reaction mixture. The activity of the recombinant protein was strongly inhibited in the presence of adenosine (Figure 5A), and inhibition by adenosine was observed with all the nucleotides used as substrate in the assay (Figure 5B).

### 2.3. Gene Expression

**Figure 6:**
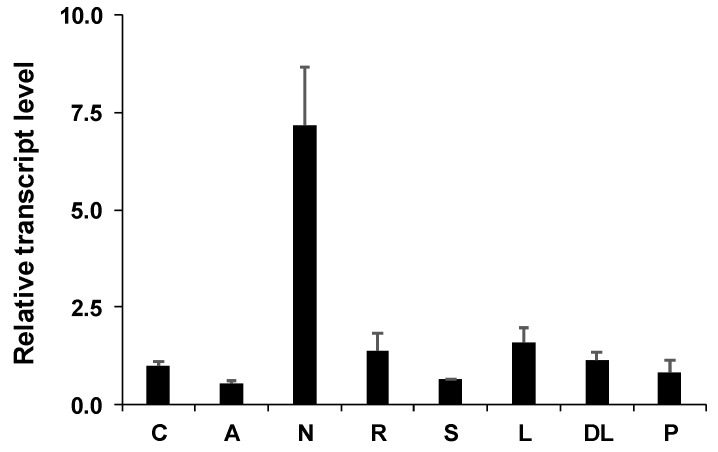
Expression pattern of *PvNTD2* in different tissues of *Phaseolus vulgaris* plants. The relative expression level was normalised using a geometric mean of two reference genes. Cotyledons (C) and embryonic axes (A) from seedlings at 5 d of development, nodules (N), roots (R), stems (S), mature leaves (L), developing leaves (DL), and mature pods (P) from adult plants. Values are mean ± SE of three independent experiments.

Expression level of *PvNTD2* was analysed in several tissues from common bean plants including cotyledons and embryonic axes from seedlings, different tissues from twenty-eight-day-old nodulated common bean plants (in the vegetative stage), and in pods of plants in the reproductive stage (Figure 6). *PvNTD2* transcript was particularly high in nodules, whereas it reached similar levels in all the other tissues.

**Figure 7:**
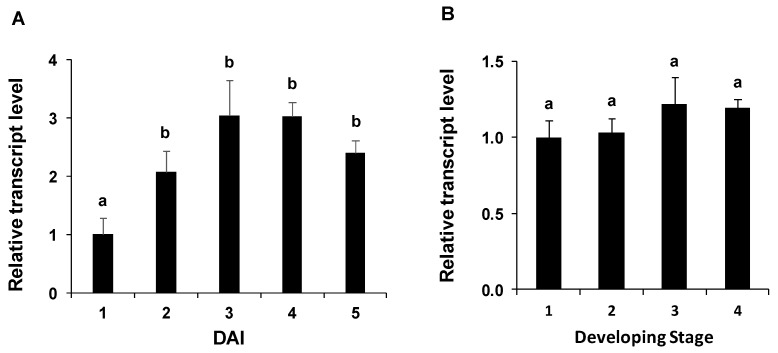
Expression pattern of *PvNTD2* in different nutrient mobilization processes. (**A**) *PvNTD2* gene expression analysis was performed using qRT-PCR on total RNA samples extracted from embryonic axes of the common bean at the days after imbibition (DAI) indicated. (**B**) Relative expression levels of *PvNTD2* during leaf ontogeny. The fourth trifoliate leaves were obtained from plants at the vegetative stage and classified according to the length of the middle leaflet: stage 1 (5–20 cm), stage 2 (20–40 cm), stage 3 (40–60 cm), and stage 4 (more than 60 cm). Different letters indicate statistically significant differences according to Bonferroni analysis (*p* ≤ 0.05).

The expression of *PvNTD2* was analysed in physiological conditions with high nutrient mobilization such as embryonic axes development and leaf development (Figure 7). The expression of *PvNTD2* increased significantly at 3 days after imbibition (DAI) in whole embryonic axes during germination and postgerminative growth (Figure 7A), whereas it remained very similar during leaf ontogeny (Figure 7B).

**Figure 8:**
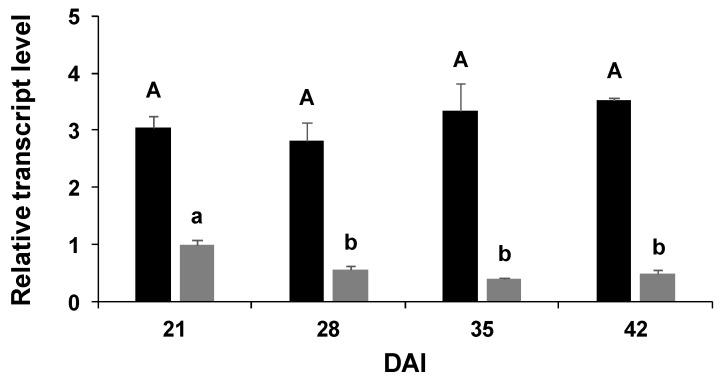
Expression patterns of *PvNTD1* (grey) and *PvNTD2* (black) during the development of nodules of the common bean. The relative expression level was normalised using a geometric mean of two reference genes. Values are mean ± SE of three biological replicates, with three technical replicates per experiment. All the values were normalised to the values of *PvNTD1* expression at 21 days after inoculation. Difference letters indicate statistically significant differences according to Bonferroni analysis (*p* ≤ 0.05).

Since the expression of *PvNTD2* was higher in nodules than in all the other tissues, we analysed its expression during nodules development and compared its expression to that of *PvNTD1* (Figure 8). *PvNTD2* was expressed at higher levels and presented a different expression pattern than *PvNTD1* in this organ. *PvNTD1* expression was higher in immature nodules and decreased during nodule development, whereas that of *PvNTD2* was similar during all the developing stages analysed (Figure 8).

## 3. Discussion

Despite the advantages of genome sequencing, it is not clear which gene or genes are responsible for encoding the protein or proteins involved in dephosphorylation of nucleotides. It is also unknown if the enzyme or enzymes show specificity for some nucleotides, if they can distinguish between purines or pyrimidines, or if they can dephosphorylate in vivo all the nucleotides. *PvNTD2* was identified as the gene from common bean with the highest identity to the previously identified and characterised *PvNTD1* [13]. PvNTD2 has the characteristic domains of the HAD (haloacid dehalogenase-like hydrolases) superfamily, which includes, among other proteins, hydrolases, phosphatases, nucleotidases, and several phosphotransferases [11]. Apart from these motifs, the members of the HAD superfamily have a high sequence divergence, and the catalysed reactions and substrate specificities are difficult to predict and must be determined empirically [28]. *PvNTD2* encodes a protein that can use both purine and pyrimidine nucleotides as substrate.

PvNTD1 shares some characteristics with PvNTD2. Both are insensitive to molybdate, a common inhibitor of acid phosphatases [14], have reduced activity with AMP as substrate compared to the other nucleotides, and are specifically inhibited by adenosine. Although this feature is shared with PvNTD1, the inhibition was higher in PvNTD2 than in PvNTD1 [13]. PvNTD2 also shared with PvNTD1 the tendency to form reducing-resistant dimers. Western blot analysis revealed the possibility of PvNTD2 forming dimers in reducing SDS-PAGE, and this behaviour was observed as well in plant-purified PvNTD1, which required urea treatment to fully denature the protein to monomers [12,13].

Nucleotide synthesis by salvage pathways is energetically favourable for cells; therefore, if bases or nucleosides are abundant, these are recycled to synthesise nucleotides. In plant cells, the levels of free nucleosides and bases are much lower than those of nucleotides [29]. It has been postulated that the balance between synthesis and degradation of nucleotides could be regulated by the ratio between phosphatase and kinase activities [11]. If the kinase predominates against the phosphatase, the synthesis of nucleotides would be favoured against degradation, resulting in anabolic processes instead of catabolic. Our results suggest that adenosine could have a crucial role in this balance, inhibiting the degradation of nucleotides when its concentration increases. The kinetic constants also could support this role for AMP/adenosine. Among the substrates analysed, a higher affinity was found for AMP, with a low value of K_m_ and high catalytic efficiency (V_max_/K_m_), than with the nucleotides with the highest specific activity (UMP, TMP, and XMP).

Nucleotidases can be involved in processes in which purine nucleotide metabolism can have a role for nutrient mobilization such as seeding [2,3] or leaf [4] development. A source for nucleotides can be nucleic acids, compounds that, during nutrient mobilization, must be degraded to relocate the degradation products to the new developing tissues. The expression of *PvNTD2* was induced in embryonic axes in concordance to the increase in nuclease [3,30] and nucleotidase activities, and *PvNTD1* expression [13], suggesting that both nucleotidases participate coordinately in this process. During leaf ontogeny, clear differences in the expression of *PvNTD1* and *PvNTD2* were found: *PvNTD1* expression was high in young leaves and decreased during leaf ontogeny [13], whereas the expression of *PvNTD2* was maintained during the whole process (Figure 7), suggesting different roles for both proteins.

The expression of *PvNTD2* was higher in nodules than in the rest of the tissues. The common bean is a ureidic legume that transports the fixed nitrogen to the upper part of the plant as ureides. Ureides are synthesized from the catabolism of purines; therefore, one or several nucleotidase/s must be involved [1]. The high expression of *PvNTD2* in nodules could suggest a role of PvNTD2 in ureide synthesis. However, its expression pattern in nodules does not correlate with the pattern of nitrogen fixation in common bean [31]. Nevertheless, we cannot discard a role of PvNTD2 in de novo ureides synthesis, since this lack of strict correlation has been also observed for the first enzyme committed to de novo ureides synthesis in nodules [31]. Two phosphatase activities purified from nodules from ureidic legumes have been reported: one of common bean [32] and another one of soybean [15]. PvNTD2 is clearly a different protein with a different activity than that purified by Garcia and colleagues from common bean [32], since the latter has a molecular weight of 120 kDa and is sensitive to molybdate. On the other side, the enzyme from soybean is a 70 kDa protein whose behaviour in the presence of molybdate is not reported [15]. The authors indicated that the soybean protein is the 5’-nucleotidase involved in the conversion of nucleotides to nucleosides in the pathway leading to ureide synthesis in nodules. The activity of the soybean enzyme with pyrimidine nucleotides was not reported, and it was similar with all the purine nucleotides tested, including AMP [15,16]. PvNTD2 can hydrolyse both purine and pyrimidine nucleotides with similar in vitro activity, with the exception of AMP. *PvNTD2* shows some identity with the gene encoding the enzyme purified in soybean; nevertheless, in this organism, there are other sequences with higher identity to *PvNTD2* (Table S1), which suggests that *PvNTD2* and the gene encoding the nodule soybean nucleotidase are not orthologous. Furthermore, PvNTD2 was predicted to be in the apoplast, which does not seem to fit with a role as the main enzyme involved in ureide biosynthesis. However, the probability of this location is 0.73, and DeepLoc prediction has an accuracy of 78% so that another location cannot be totally discarded. A different function to ureide biosynthesis during nitrogen fixation is also plausible.

Nitrogen-fixing legumes demand more P than non-nodulating plants for optimal functioning, and under P deficiency, the growth of these nitrogen-fixing legumes is retarded [33]. It has been proposed that the level of P in the nodules is determinant for the symbiotic efficiency, and several mechanisms have been proposed for the maintenance of P in nodules [33]. Therefore, PvNTD2 could play a role supplying P to the nodule to fulfil this special requirement.

To summarize, the nucleotidase described in this work can use in vitro all the nucleoside monophosphates as substrate, and it is insensitive to molybdate. In addition, *PvNTD2* is expressed in situations with high nucleotide metabolism. All of this supports the role of this protein as a nucleotidase. Like *PvNTD1, PvNTD2* is expressed in all the tissues analysed, although the expression pattern of both genes shows clear differences, mainly in leaf ontogeny and nodules. The high expression of *PvNTD2* in nodules and its maintenance during development in contraposition to *PvNTD1* expression, which levels are lower and decrease during nodule development, support a different role for both *PvNTD1* and *PvNTD2* in nodules of the common bean. Whether this role is related to the de novo synthesis of ureides or not needs further investigation.

## 4. Materials and Methods

### 4.1. Plant Material and Growth Condition

Common bean (*Phaseolus vulgaris* L. cv. Great Northern) seeds were sterilised in ethanol (30 s) and sodium hypochlorite 0.2% (*w*/*v*) (5 min). The surface-sterilised seeds were washed repeatedly with distilled water and then placed in Petri dishes (120 mm diameter) with wet paper in sterile conditions.

The material from seedlings up to 6 d after start of imbibition were obtained from seedlings maintained in the Petri dishes with regular addition of distilled water. To obtain the material from adult plants, germinated seedlings were sown on pots 3 d after imbibition, and the plants were inoculated with *Rhizobium leguminosarum* ISP14 and cultivated and maintained as indicated elsewhere [34]. The following samples were collected from plants after 28 d of growth: roots after careful removal of nodules, mature and immature leaves, and shoots after removal of leaves and nodules. The flowers and pods were obtained from plants in the reproductive stage. Studies during the development of the leaves and nodules were performed as described [34].

### 4.2. Cloning into the pET30b(+) Expression Vector

The coding region of *PvNTD2* cDNA lacking the signal peptide was obtained by PCR from cDNA from senescing leaves from common bean using the primers NTD400Pv01 and NTD400Pv02 containing restriction sites for *Nco*I and *Xho*I, respectively (Table S2). The PCR product was cloned and transformed into *Escherichia coli* DH5αF’. The insert was digested with *Nco*I and *Xho*I, cloned into the vector pET30b(+), and transformed into *E. coli* BL21 (DE3) cells (Novagen). The pET30b(+) allows the expression of the recombinant protein fused to a His tag at both ends. The final construct was sequenced to confirm the absence of mutations.

### 4.3. Expression and Purification of the Recombinant Protein

The transformed cells were grown in 200 mL of LB medium supplemented with 50 mg/L kanamycin at 37 °C until an absorbance of 0.5 at 600 nm was reached. Isopropyl-β-d-thiogalactopyranoside (IPTG) was added at a final concentration of 1 mM to the cultures, and the expression of the heterologous protein was induced at 37 ° C for 2 h. Afterwards, the cells were harvested by centrifugation at 2000× *g* and 4 °C for 10 min, and the pellet was stored at −20 °C.

The pellet was resuspended in 4 mL of lysis buffer (50 mM TRIS-HCl (pH 7.8), 1 mM MgCl_2_, 150 mM NaCl, 0.02% (*v*/*v*) Tween 20, and 10% (*v*/*v*) glycerol), and cells were lysed by sonication on a Vibra Cell (Sonics and Materials Inc., Newton, MA, USA) using 3 pulses of 90 W and 5 s each, keeping the samples on ice. After sonication, the homogenate was centrifuged at 12000× *g* for 15 min at 4 °C. The supernatant was used as crude extract for further purification. The pellet was resuspended in 4 mL of lysis buffer to analyse the insoluble fraction corresponding to inclusion bodies. The crude extract was passed through a Nickel Chelating Sepharose column (1 mL, GE Healthcare, Uppsala, Sweden) equilibrated with lysis buffer. The column was washed with 5 mL of lysis buffer, followed by a second wash with 5 mL of lysis buffer supplemented with 25 mM imidazole. The protein was eluted with 3 mL of the same buffer supplemented with 200 mM imidazole.

### 4.4. Gel Electrophoresis, Western Blot Analysis, and Protein Stain

Proteins were mixed with denaturing buffer and boiled for 5 min and fractionated in 10% SDS-PAGE gels by using a Mini PROTEAN III system (Bio-Rad). After separation, proteins were electro-transferred to a polyvinylidene fluoride (PVDF) membrane (Sigma-Aldrich, St Louis, MI, USA). To detect His-tagged protein, blots were incubated with a monoclonal anti-polyhistidine antibody (H1029, Sigma-Aldrich) at 1:10,000. Anti-mouse IgG, alkaline phosphatase conjugated (A3562, Sigma-Aldrich), was used as secondary antibody at a 1:12,500 dilution, and the immunoreaction was developed with 5-bromo-4-chloro-3-indolyl phosphate p-toluidine salt (125 mg mL^−1^) and nitro-blue tetrazolium chloride (250 mg mL^−1^) as substrates. Silver staining was performed using the Silver Staining kit (Sigma-Aldrich).

### 4.5. Enzymatic Activity

Phosphatase activity was determined as previously indicated [12]. The phosphatase activity was calculated as the production of phosphate along time in reaction mixtures with several phosphorylated compounds. Unless otherwise stated, the standard reaction mixture for recombinant PvNTD2 purified from *E. coli* contained 50 mM TES-NaOH (pH 7), 1 mM MgCl_2_, 2 mM substrate, and an adequate amount of enzyme. The reactions were initiated by the addition of the enzyme and were performed at 37 °C. Aliquots of 0.2 mL were extracted several times, and the amount of phosphate was determined. One unit of enzyme is defined as the amount of enzyme that catalyses the formation of 1 µmol of phosphate per minute.

### 4.6. RNA Isolation and cDNA Synthesis

Total RNA was isolated from several tissues using the NZYol Reagent (NZYTECH) according to the manufacturer’s instructions, except that an additional LiCl precipitation step was included at the end to improve the RNA quality. A 2 µg sample of total RNA was treated with RNAase-free DNAseI (NEB) at 37 °C for 30 min to eliminate any traces of genomic DNA. First-strand cDNA synthesis was performed with DNAse-treated RNA using a Revert Aid reverse transcriptase (ThermoFisher) with random hexamer primers.

### 4.7. Real-Time PCR

Quantitative RT-PCR (qRT-PCR) was carried out with an iCycler iQ system using the iQ SYBRGreen supermix (Bio-Rad) and the gene-specific primers specified in Table S2. The PCR program consisted of an initial denaturation of 5 min at 95 °C, followed by 40 cycles of 15 s at 95 °C, 30 s at 60 °C, and 30 s at 72 °C. After the final cycle, a melting curve analysis was performed over a temperature range of 60–100 °C by increments of 0.5 °C in order to verify the reaction specificity. Results were normalised using the geometric mean of two control genes (ubiquitin and actin-2) using the 2^−ΔΔCT^ method [35].

### 4.8. Analytical Determination

Protein concentration was determined by the Bradford method [36] using bovine serum albumin as standard and the Bio-Rad system.

### 4.9. Statistical Analyses

All results are means of three independent experiments with three technical replicates per experiment. Values are mean ± SE. Statistical analyses were performed with SPSS Statistics, version 17.0, using unifactorial ANOVAs. Homoscedasticity was determined using the Levene test, and Bonferroni statistics were applied for variables with homogeneity of variance.

## Figures and Tables

**Table 1:** Kinetic properties of purified PvNTD2 with several nucleotides. Activity was assayed under standard conditions, and the values for K_m_ and V_max_ were calculated by Hanes–Woolf plots.

Substrate	K_m_ (mM)	V_max_ (U/mg)	V_max_/K_m_
AMP	0.011	1.46	132
UMP	0.039	4.00	103
TMP	0.063	4.08	64
XMP	0.095	4.22	44

## Supplementary Materials

The following are available online at https://www.mdpi.com/2223-7747/9/2/171/s1, Table S1: Sequences with the highest homology after searching in NCBI database using tblastn, Table S2: Primers used in this study.
