# Supplementary material for: Biochemical and Molecular Characterization of PvNTD2, a Nucleotidase Highly Expressed in Nodules from Phaseolus vulgaris
Source: Plants (Basel). 2020 Feb 1;9(2):171. doi: 10.3390/plants9020171 (PMC7076459; doi:10.3390/plants9020171)
Supplement: Supplementary file 1 [file plants-09-00171-s001.pdf]

Supplementary Table S1. Sequences with the highest homology after searching in NCBI database using tblastn.

Accession	Description	E-value	Indent (%)
XM_014635331.2	PREDICTED: Vigna radiata var. radiata acid phosphatase 1	1e-162	91
XM_017576152.1	PREDICTED: Vigna angularis acid phosphatase 1	1e-161	91
XM_028054234.1	PREDICTED: Vigna unguiculata acid phosphatase 1	1e-159	89
NM_001253125.1	Glycine max acid phosphatase 1-like	4e-149	87
XM_028349321.1	Glycine soja acid phosphatase 1-like	2e-148	87
XM_004511071.3	Cicer arietinum acid phosphatase 1-like	5e-140	80
AK336967.1	Lotus japonicus cDNA	5e-139	80
XM_027500120.1	PREDICTED: Abrus precatorius acid phosphatase 1	4e-138	82
XM_028389016.1	Glycine soja acid phosphatase 1-like	1e-137	81
XM_003531750.4	PREDICTED: Glycine max acid phosphatase 1	2e-137	81
XM_020355322.1	PREDICTED: Cajanus cajan acid phosphatase 1-like	2e-132	83
XM_019609406.1	PREDICTED: Lupinus angustifolius acid phosphatase 1-like	3e-128	73
XM_003628201.3	PREDICTED: Medicago truncatula acid phosphatase 1	3e-126	74
BT143084.1	Medicago truncatula clone JCVI-FLMt-4L7 unknown mRNA	7e-126	75

Supplementary Table S2. Primers used in this study. Sequences read from 5′ to 3′.

	Gene	Forward
pET30 cloning	*NTD400Pv01*	TCTTCCATGGATTCCGATTCTCCGAGTGCG
	NTD400Pv02	TGAGCTCGAGCTAAGAGATGTAATACATGG GATTTGG
qRT-PCR	*qNTD1-D1*	GAAGTGGTCGGGAATTTGTCG
	qNTD1-R1	ATCTCAAACCCGAATCCAATGTC
	qPvNTD2-F	TGAAACTCAGTGACGATGGCAAA
	qPvNTD2-R	CTCTACCCAATCGTTGAATTTCTCA
	qUBQ-F3	TACATGCGATCTTGGACTGGC
	qUBQ-R3	GGGGCTTTTCTGGGTAGTCT
	qActin-2-F3	GCAATTCAGGCTGTCTTGTCTTTGT
	qActin-2-F3	TAAATCACGGCCAGCAAGATCC
